# Joint genome-wide association and transcriptome sequencing reveals a complex polygenic network underlying hypocotyl elongation in rapeseed (***Brassica napus L.***)

**DOI:** 10.1038/srep41561

**Published:** 2017-01-31

**Authors:** Xiang Luo, Zhifei Xue, Chaozhi Ma, Kaining Hu, Ziru Zeng, Shengwei Dou, Jinxing Tu, Jinxiong Shen, Bin Yi, Tingdong Fu

**Affiliations:** 1 National Key Laboratory of Crop Genetic Improvement, National Center of Rapeseed Improvement in Wuhan, Huazhong Agricultural University, Wuhan 430070, P.R. China

## Abstract

Hypocotyl elongation is considered an important typical seedling trait contributing directly to an increase in and stabilization of the yield in *Brassica napus*, but its molecular genetic mechanism is poorly understood. In the present study, hypocotyl lengths of 210 lines were measured in an illuminated culture room. A genome-wide association study (GWAS) was performed with 23,435 single nucleotide polymorphisms (SNPs) for hypocotyl length. Three lines with long hypocotyl length and three lines with short hypocotyl length from one doubled haploid line (DH) population were used for transcriptome sequencing. A GWAS followed by transcriptome analysis identified 29 differentially expressed genes associated with significant SNPs in *B. napus*. These genes regulate hypocotyl elongation by mediating flowering morphogenesis, circadian clock, hormone biosynthesis, or important metabolic signaling pathways. Among these genes, *BnaC07g46770D* negatively regulates hypocotyl elongation directly, as well as flowering time. Our results indicate that a joint GWAS and transcriptome analysis has significant potential for identifying the genes responsible for hypocotyl elongation; The extension of hypocotyl is a complex biological process regulated by a polygenic network.

Rapeseed (*Brassica napus L.*, 2n = 4x = 38; genome AACC) is one of the most important oilseed crops in the world and the genetic control of yield and yield-related traits has been studied extensively. However, less attention has been focused on elucidating the genetic mechanism of seedling traits. Well-developed seedling traits can contribute directly to an increase in and stabilization of yield and its heterosis, mineral nutrient absorption, drought tolerance, and salinity tolerance in crops^1^,^2^,^3^,^4^,^5^,^6^,^7^,^8^,^9^,^10^. Thus, understanding the seedling traits will be conducive to breeding programs in *B. napus*.

In rapeseed, early seedling development traits have been shown to significantly correlate with agronomic traits^2^. Many heterosis-related quantitative trait loci (QTLs) for seedling traits in *B. napus* are common with yield-related QTLs^1^. Several promising positional and functional candidate genes have been associated with germination speed, absolute germination rate, and radicle growth in *B. napus*^6^. The number of loci detected for 14 seedling development traits, and candidate genes *GER1, AILP1, PECT,* and *FBP* were reported to strongly relate to the seedling development traits in *B. napus*^7^. Hypocotyl elongation is thought to be an importantly typical seedling trait in plants. However, almost all studies on hypocotyl elongation have focused on *Arabidopsis.* Global transcriptome analysis has revealed circadian regulation of key pathways in rhythmic hypocotyl elongation^11^. The transcriptional regulators *CIRCADIAN CLOCK ASSOCIATED1 (CCA1), EARLY FLOWERING3 (ELF3), ELF4,* and *LUX ARRHYTHMO (LUX)* appear to link the circadian clock to diurnal control of hypocotyl growth^12^,^13^. Therefore, hypocotyl elongation has a fiercely complex genetic mechanism, but little knowledge is available about hypocotyl elongation in *B. napus*.

Expressed sequence tag (EST) sequence data, array analysis, amplicon resequencing, sequences, and next-generation sequencing technologies efficiently allow genome-wide association studies (GWASs) and transcriptome analysis to be novel strategies for dissecting complex traits in plants^14^,^15^,^16^,^17^,^18^,^19^,^20^,^21^. In the present study, a GWAS for hypocotyl elongation was carried out with a panel of 210 *B. napus* accessions genotyped for 23,435 SNPs. We also performed transcriptome sequencing of long and short hypocotyl phenotypes. The main objectives of this study were to obtain a better understanding of hypocotyl elongation and its relationship with yield-related traits or heterosis, dissect the genetic basis of hypocotyl elongation by combining GWAS and transcriptome analysis, and perform Gene Ontology (GO) and KEGG pathway analysis for associated genes in *B. napus*.

## Results

### Phenotypic variations and correlation analysis

**Figure 1:**
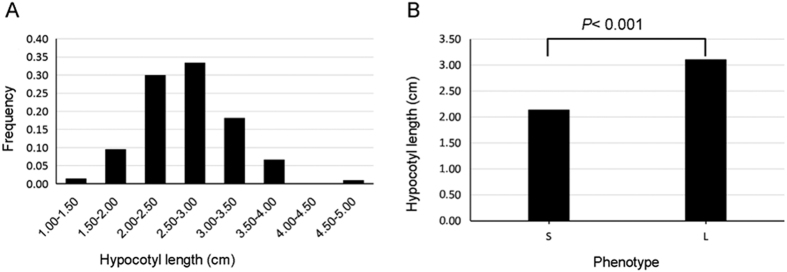
Phenotypic variation in hypocotyl length. (**A**) Frequency of phenotypic variation in 210 accessions. (**B**) Comparison of two phenotypes by *t*-test.

Extensive phenotypic variations in hypocotyl elongation were observed in the 210 rapeseed lines (Fig. 1A and Supplementary Tables S1 and S2). The hypocotyl elongation of the lines was normally distributed (average = 2.66, range 1.38 to 4.81), and 63.33% of hypocotyl elongation values were between 2.00 and 3.00.

The correlation coefficients between hypocotyl elongation and yield-related traits showed that hypocotyl elongation positive correlated with seed yield per plant (0.29) and biomass yield per plant (0.21) at *P* = 0.01 and plant height (0.19) at *P* = 0.05 (Supplementary Table S3). Linear regression analysis of the correlated traits indicated that hypocotyls elongation can explain 3.28% of the total seed yield per plant (*P* < 0.05), 4.49% of the total biomass yield per plant (*P* < 0.01), 3.59% of the total plant height (*P* < 0.05), respectively.

### Genetic diversity, population structure, and relative kinship analysis

**Figure 2:**
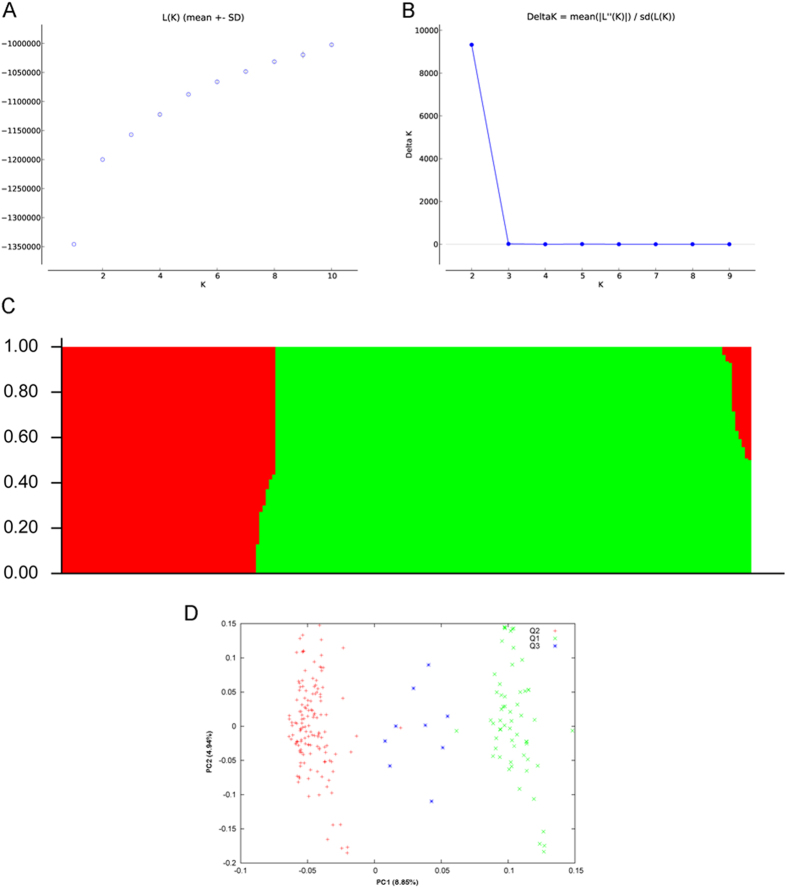
Analysis of the population structure of 210 rapeseed accessions using *STRUCTURE*. (**A**) Estimated L(K) of possible clusters (k) from 1 to 10. (**B**) Delta K based on the rate of change of L(K) between successive K values. (**C**) Population structure based on k = 2. Red represents subgroup Q1; green represents subgroup Q2. (**D**) Principal component (PC) analysis.

**Figure 3:**
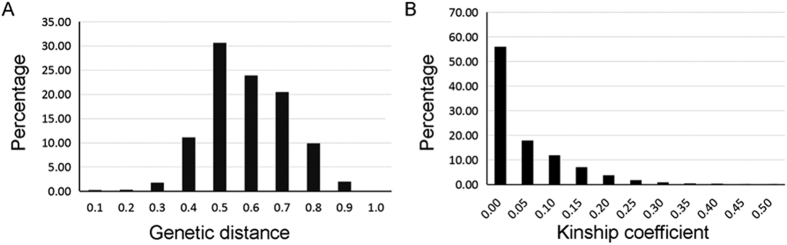
Genetic distance and kinship coefficient analysis between pairs of accessions.

The genetic diversity and population structure of the 210 accessions were analyzed using 5,334 SNPs (Supplementary Table S4). Clustering inference showed that the most significant change in likelihood occurred when K increased from 2 to 3, and the highest Δk value was observed at K = 2 (Fig. 2A–C). Considering the probability of membership threshold of 0.70, 61 and 140 accessions were assigned to subgroups Q1 and Q2, respectively (Supplementary Table S1). The remaining nine accessions were assigned to a mixed group (Q3). The PCA also provided a pattern for the genetic structure of the GWAS population (Fig. 2D). The top two principal components clearly separated these subpopulations and explained 8.85% and 4.94% of the total SNP variations in the rapeseed panel, respectively. All of the parameters suggest that the three-group model (subgroups Q1, Q2, and Q3) sufficiently explained the genetic structure among the 210 accessions. The mean genetic distance (GD) between lines was 0.54, and 74.85% of pairs had a GD ranging from 0.5 to 0.7 (Fig. 3A). The average kinship coefficient identity by descent (IBD) within the total diversity set was 0.06 (Fig. 3B). A total of 55.93% of the pairwise kinship estimates were equal to 0, and 17.85% of pairwise kinship coefficients varied from 0 (excluding 0) to 0.05.

### LD analysis

All 23,435 SNPs in the total panel were used for LD analysis. The distributions of *r*^*2*^ with respect to the physical distance from each chromosome are shown in the Supplementary data (Fig. S1 and Supplementary Table S5). As expected, the mean *r*^*2*^ between 0 and 500 kb decreased rapidly and continuously, followed by much slower decay at increased physical distance for both the A genome and C genome. The overall LD decay distance was 893.84 Kb when the *r*^2^ cutoff was set to 0.1. The rate of LD decay varied over different chromosomes in both the A genome and C genome, with the shortest LD decays of 459.03 kb on chromosome A07 and 602.91 kb on chromosome C08 and the longest LD decays of 968.17 kb on chromosome A09 and 3,190.79 kb on chromosome C09. Obviously, the LD of the A genome decayed significantly faster than the LD of the C genome.

### Association mapping and candidate gene prediction

**Figure 4:**
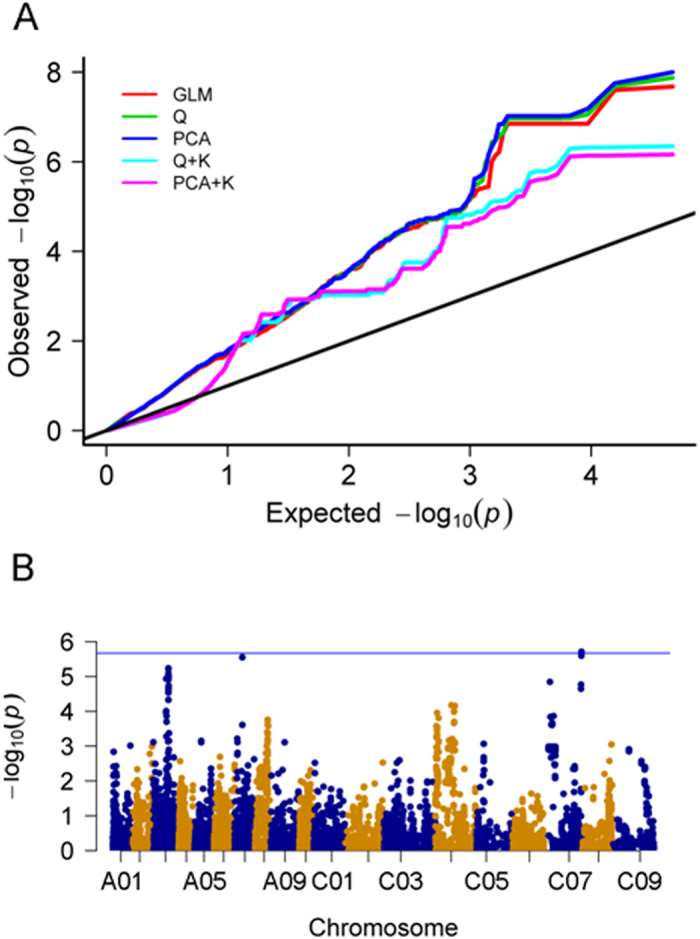
Association analyses of hypocotyl elongation. (**A**) Quantile–quantile plots of estimated −log_10_(*P*) from the association analysis of hypocotyl elongation. The black line represents expected *P*-values with no associations. The red line represents observed *P*-values using the GLM model. The green line represents observed *P*-values using the Q model. The blue line represents observed *P*-values using the PCA model. The cyan line represents observed *P*-values using the Q + K model. The pink line represents observed *P*-values using the PCA + K model (color figure online). (**B**) Manhattan and quantile–quantile plots generated from the genome-wide association analysis of hypocotyl elongation. The blue horizontal line depicts the Bonferroni significance threshold (2.13 E-6).

**Figure 5:**
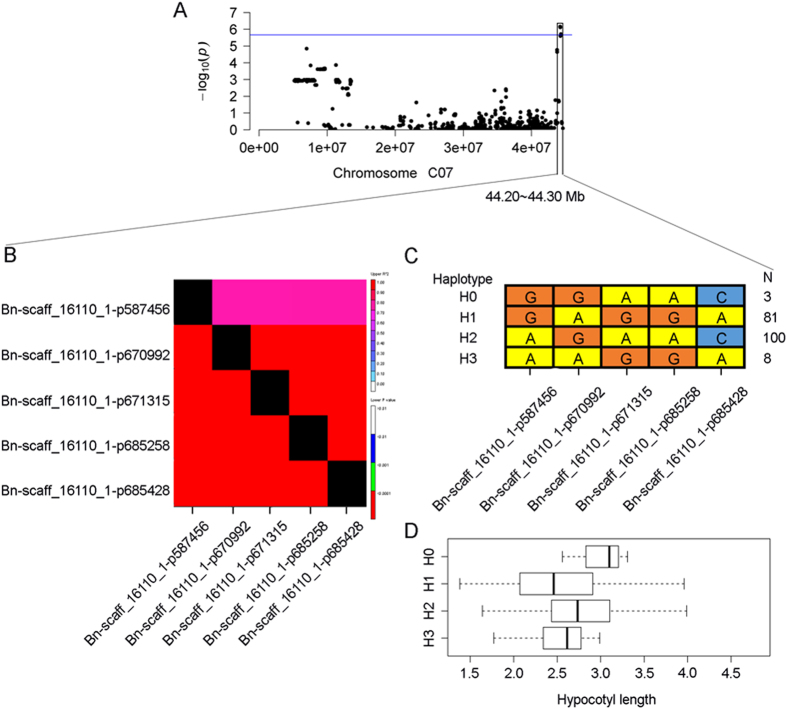
LD and haplotype analysis for five associated SNPs. (**A**) Association peak on chromosome C07. (**B**) LD analysis among the associated SNPs. (**C**) Haplotype analysis with associated SNPs in the population. (**D**) Phenotypic variation of hypocotyl length in each haplotype.

Total 23,435 polymorphisms with minor allele frequency (MAF) ≥ 0.05 were selected for association mapping of hypocotyl elongation using the BLUP value across multiple replications (Supplementary Table S1). Model comparison analyses indicated that *P*-values from the PCA + K model were nearer the expected *P-*values than those of the GLM, Q, PCA, and Q + K models (Fig. 4A). Thus, the PCA + K model was selected for association mapping of hypocotyl elongation. Five SNPs on C07 were highly significantly associated with hypocotyl elongation at *P* < 2.13 E−06, with a FDR of 1.0% (Fig. 4B and Table 1). All detected SNPs were located between 42.15 and 42.25 Mb on C07 and could explain 4.82% of the total phenotypic variance. Thus, the development of hypocotyl is controlled by a minor-effect polygene. In LD analyses, the *r*^2^ values were > 0.79 for all pairs of associated SNPs, suggesting that the associated SNPs were in high LD with each other (Fig. 5A and B).

According to the associated SNP variations, four haplotypes (H0, H1, H2, and H3) were identified from these *B. napus* accessions (Fig. 5C). H0, H1, H2, and H3 were observed in 3, 81, 100, and 8 lines, respectively. Approximately 94.27% of accessions comprised H1 and H2. Therefore, H0 and H3 are rare variations, whereas H1 and H2 are conserved by artificial selection during the genetic improvement of modern *B. napus* breeding accessions. Further analysis showed that H0, H1, H2, and H3 have mean hypocotyl elongations of 2.99, 2.52, 2.79, and 2.52, respectively (Fig. 5D). H2 had a significantly (*P* = 0.001) greater hypocotyl elongation than H1. H0 and H3 were not analyzed because they are extremely rare. Thus, H2 may be a favorable haplotype and facilitate the selection of better genotypes for hypocotyl elongation in breeding *B. napus*.

Candidate genes were predicted along the ~100 Kb region between two associated SNPs (Bn-scaff_16110_1-p685428 and Bn-scaff_16110_1-p587456) according to the newly released *B. napus* genome sequence^22^. Only five genes (*BnaC07g46740D, BnaC07g46760D, BnaC07g46770D, BnaC07g46780D,* and *BnaC07g46800D*) were detected in the candidate region (Supplementary Table S6). Of these genes, *BnaC07g46770D* was previously identified to regulate the flowering time in rapeseed^23^. The closest distance between *BnaC07g46770D* and a significant SNP (Bn-scaff_16110_1-p670992) was 34 Kb. Considering the LD decay of 754.95 Kb in C07, candidate genes were also predicted in the region between 754.95 Kb upstream and downstream of the associated peak; 196 genes were obtained in the enlarged candidate region (Supplementary Table S6). All of the genes were blasted against *A. thaliana* genome data, but none of the predicted genes were homologous to the genes directly controlling hypocotyl elongation in *Arabidopsis*.

### Transcriptome sequencing analysis

The DH-6004 population had considerably variable flowering time when grown at Hezheng, Gansu province, in the 2015 growing season and Wuhan, Hubei province, in the 2015–2016 growing season. Three lines with extremely early-flowering and three lines with extremely late-flowering exhibited long hypocotyls and short hypocotyls, respectively. The mean hypocotyl elongation in the S and L groups was 2.14 and 3.11, respectively (*P* < 0.001, *t* test; Fig. 1B and Supplementary Table S1). Haplotype analyses indicated that DH2 and DH3 in S group showed H1, and DH4, DH5 and DH6 in L group showed H2. The DH1 could not been distributed to any of the four haplotypes as it possessed heterozygosity loci. RNA from the three S lines and three L lines was pooled with two biological replications to generate S1, S2, L1, and L2. A total of 26.82, 54.07, 55.43, and 28.87 million raw sequence reads were generated from the four libraries (Supplementary Table S7). After removing low-quality reads and adaptor sequences, 23.61, 49.17, 49.97, and 25.88 million clean reads were obtained for S1, S2, L1, and L2, respectively. More than 70% of the reads were successfully mapped to the reference genome; the unique and multiple reads that aligned with the genome accounted for 73.09% in L2 to 86.49% in S2.

Of the 196 genes located within the candidate region determined by the GWAS analysis, 29 significant DEGs were identified in the two groups based on the criteria |log_2_(L/S)| ≥ 1 and *P* < 0.05 (Table 2). Compared to the S group, 16 (53.33%) DEGs were up-regulated and 13 (46.67%) DEGs down-regulated in the L group.

### Functional classification of DEGs

To monitor the gene expression pattern, GO enrichment analysis of DEGs was performed for two genotypes (Fig. S2). The 29 DEGs were finally classified into 10, 6, and 19 main GO categories according to the cellular component (CC), molecular function (MF), and biological process (BP), respectively. The CC categories, such as cell, cell part, and organelle, were overrepresented. Most of the DEGs function in catalytic activity and binding. The BP category occurring in metabolic processes was significantly overrepresented and included approximately 83% of the DEGs. Further cluster analysis according to BP indicated that eight DEGs (*BnaC07g45520D, BnaC07g45710D, BnaC07g45720D, BnaC07g46090D, BnaC07g46630D, BnaC07g46660D, BnaC07g46770D,* and *BnaC07g47470D*) were associated with the response to hormone and flower morphogenesis (Supplementary Table S8).

To explore the function of DEGs in the biosynthesis and metabolite pathways, KEGG pathway analysis was performed in two phenotypic groups (Supplementary Table S9). Six DEGs (*BnaC07g45590D, BnaC07g45710D, BnaC07g46060D, BnaC07g46560D, BnaC07g46660D,* and *BnaC07g47470D*) acted in the 30 pathways by encoding corresponding enzymes. For example, *BnaC07g46060D* and *BnaC07g46560D* regulate the lignins and phenylpropanoid biosynthesis in phenylpropanoid metabolic pathways by encoding dehydrogenase and lactoperoxidase, respectively. Furthermore, *BnaC07g46060D* and *BnaC07g47470D* participate in glycolysis/gluconeogenesis and nitrogen metabolism and carbon fixation in photosynthetic organisms by encoding dehydrogenase and aldolase, respectively. A global examination of gene expression demonstrated that genes encoding dehydrogenase regulate the phenylpropanoid and lignin biosynthetic pathways and are clock-controlled in the same manner as the pathways involved in the assimilation of mineral nutrients and carbon fixation in the process of photosynthesis^11^. However, no direct evidence is available regarding the detected genes regulating metabolic pathways to affect hypocotyl elongation in relation to circadian rhythm.

### Comparative analysis

**Figure 6:**
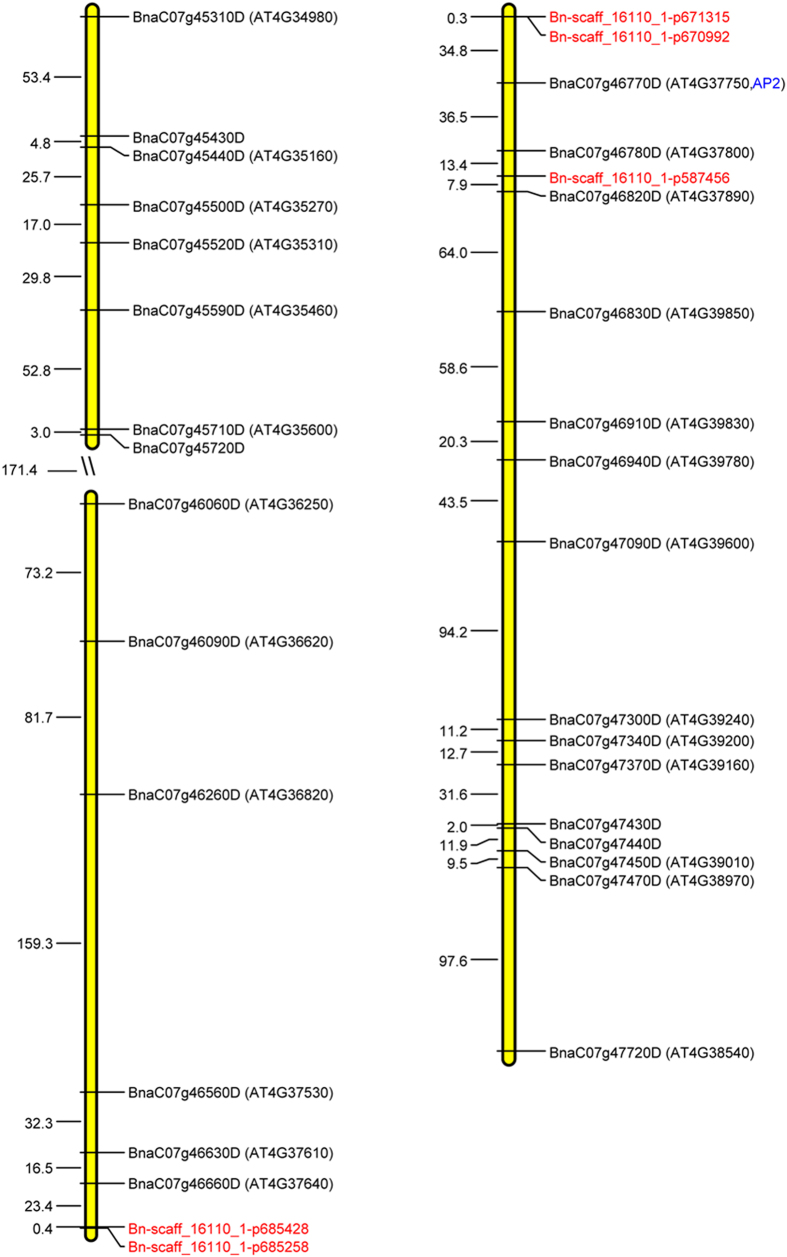
The distribution pattern of candidate genes and SNPs associated with hypocotyl elongation. The abbreviations for orthologous genes in *A. thaliana* are shown in parentheses. SNPs are marked in red. Numbers represent the relative distance in the reference genome in kilobases.

Of the 29 DEGs detected by combining transcriptome sequencing analysis and a GWAS, 26 homologous genes were identified using the *Brassica* Genome Browser database and *A. thaliana* Genome Browser database (Fig. 6 and Table 2). *BnaC07g46770D* and *BnaC07g46780D* were located within 84.7 Kb of two significant SNPs: Bn-scaff_16110_1-p670992 and Bn-scaff_16110_1-p587456. *BnaC07g46770D* was previously reported to regulate flowering time and is orthologous to *A. thaliana AP2* and *AT4G37750. AP2* belongs to the AP2/ERF gene family and is involved in plant development, in turning leaves into floral organs^24^. *AT4G37750* belongs to the AP2/EREBP gene family and directly regulates a key clock gene (*CCA1*) that provides molecular links between different signaling modules and the circadian clock^25^. *BnaC07g46780D* is orthologous to *AT4G37800*, one member of the complex endotransglucosylase/hydrolase (XTH) gene family acting within floral stages to strengthen or loosen cell walls^26^,^27^.

*BnaC07g46660D, BnaC07g46630D,* and *BnaC07g46060D* were located 23.4 Kb, 39.9 Kb, and 386.4 Kb upstream of associated SNP Bn-scaff_16110_1-p685428, and are orthologous to *AT4G37640, AT4G37610,* and *AT4G36250,* respectively*. AT4G37640* functions in a complex process of pollen germination and tube growth^28^. *AT4G37610,* which encodes *TAZ* domain protein, could act as the master clock control gene *CCA1* to regulate the organic nitrogen-responsive genes^29^. *AT4G36250* contains five TGTG sites and one HUD site and could been regulated by *TOC1(TIMING OF CAB EXPRESSION1*), which acts as a general transcriptional repressor to negatively regulate *CCA1/LHY*^30^.

*BnaC07g46830D, BnaC07g46910D, BnaC07g46940D, BnaC07g47470D,* and *BnaC07g47720D,* which are orthologous to *AT4G39850, AT4G39830, AT4G39780, AT4G38970,* and *AT4G38540,* were detected 71.9 Kb, 130.5 Kb, 150.8 Kb, 367.3 Kb, and 465.1 Kb downstream from significant SNP Bn-scaff_16110_1-p587456. *AT4G39850* and *AT4G38540* include TGTG sites and ME sites, which could also be regulated by *TOC1*^30^*. AT4G39830* showed significant changes in expression during pollen germination and tube growth and, thus, regulate the process of reproduction in *Arabidopsis*^28^*. AT4G39780* belongs to the *Arabidopsis* ERF gene family, a part of the AP2/ERF superfamily, which have important roles in the transcriptional regulation of a variety of biological processes related to growth and development, as well as various responses to environmental stimuli^31^. *AT4G38970* is expressed in the regulation of biochemical pathways during photomorphogenesis^32^. However, to date, little knowledge is available about the function of the other 15 homologous genes in *A. thaliana.*

## Discussion

Optimal seedling development of plants leads to a promising yield, and hypocotyl elongation is considered a typical seedling trait. Seedling traits measured at an early stage of development significantly correlate with agronomic traits in *B.napus*^2^. Here, we evaluated the phenotypic variation of hypocotyl elongation, which exhibited continuous variation and approximated a normal distribution. Correlation analysis indicated that hypocotyl length positively correlates with seed yield per plant, biomass yield per plant, and plant height. Five SNPs explaining 4.82% of the total phenotypic variance were highly significantly associated with hypocotyl elongation, and 196 genes were obtained in the enlarged candidate region. The results imply that hypocotyl elongation is a complex quantitative trait controlled by a minor-effect polygene.

Genome-wide association study, also known as LD mapping, has emerged as very promising strategies for understanding naturally occurring phenotypic variation^33^,^34^,^35^,^36^. Recently, more and more studies tended to identify the candidate genes by combining GWAS and linkage mapping in rice^37^, maize^38^, sunflower^39^ and wheat^40^. However, it is extremely laborious and time-consuming to develop large-scale linkage mapping populations or linkage–LD mapping populations, such as nested association mapping^41^ and multi-parent advanced generation inter-cross^42^. In rapeseed, combined SNP-trait association and transcriptome sequencing analyses successfully identified twenty-four genes associated with the resistance to *Sclerotinia* stem rot^43^. In the present study, a GWAS followed by transcriptome analysis confirmed 29 genes mainly related to circadian clock, flowering morphogenesis, hormone biosynthesis, or important metabolic signaling pathways regulating hypocotyl elongation in *B. napus*. Therefore, joint genome-wide association and transcriptome sequencing is an alternate method of dissecting the genetic and biochemical basis of hypocotyl elongation in *B. napus*.

Of the 29 genes, transcriptome sequencing assays revealed that six genes responsible for hormone (Table S8). This may correspond to the variation of hypocotyl elongation, because hormone regulates many aspects of growth and development containing hypocotyl elongation in plants. The light-mediated photomorphogenesis triggered by hormone biosynthetic factors directly affects hypocotyl elongation in *Arabidopsis*^44^. Likewise, overexpressing auxin biosynthetic genes could increase hypocotyl elongation in *Arabidopsis*^45^. In addition, six genes were detected to act in the 30 pathways by encoding corresponding enzymes, implying that these genes probably regulate the hypocotyl elongation by affecting important metabolites biosynthesis in *B.napus*. Furthermore, 25 homologs of the *Arabidopsis* genes were identified in the *B. napus* genome through homologous alignment. Among of them, *BnaC07g46770D* was previously found to directly relate to flower time^23^ and is orthologous to *A. thaliana AP2* and *AT4G37750. AP2* is involved in the development of floral organs^24^ and *AT4G37750* directly regulates a key clock gene (*CCA1*) controlled the hypocotyl elongation in *Arabidopsis*. We supposed that *BnaC07g46770D* may regulate circadian gene or floral development to affect the flowering time and hypocotyl elongation in *B.napus*, which at least partially explains the correlation between flowering time and hypocotyl elongation. Similarly, *BnaC07g46630D* is orthologous to *A. thaliana AT4G37610,* which acts as the master clock control gene *CCA1*^29^. *BnaC07g46060D, BnaC07g46830D* and *BnaC07g47720D* are orthologous to *A. thaliana AT4G36250, AT4G39850* and *AT4G38540,* respectively, regulated by *TOC1*^30^*. TOC1* is an important component of the circadian clock in *Arabidopsis* with a crucial function in the integration of light signals to control hypocotyl elongation^46^. The results indicated that these genes may affect hypocotyl elongation by interacting with circadian clock genes in *B. napus. BnaC07g47470D* is orthologous to *Arabidopsis AT4G38970* which expressed in the regulation of biochemical pathways during photomorphogenesis^32^. Photomorphogenesis is linked to photoperiod, an important challenging factors affected hypocotyl elongation by regulating cell elongation^47^. In addition, *BnaC07g46660D* and *BnaC07g46910D* are orthologous to *Arabidopsis AT4G37640* and *AT4G39830* acting within floral morphogenesis, but it needs to further study of their roles in the development of hypocotyl elongation in *B.napus*.

In summary, this study is the first to study the hypocotyl elongation by integrating GWAS and transcriptome sequencing in *B.napus*. We demonstrated that the genes mediated by circadian clock, hormone biosynthesis, floral morphogenesis, or other metabolic signaling pathways may regulate the hypocotyl elongation in *B. napus*. These findings reveal that the phenotypic variation of the hypocotyl is a complex biological process regulated by a polygenic network in *B.napus*. Over the past decade, circadian clock and hormone effects had been linked to agronomic traits in plant^48^,^49^. Hypocotyl elongation represents the best-studied model of plant circadian clock and hormone response system. Therefore, modification of these areas may have the potential for systemic effects that produce beneficial yield trait in *B.napus*.

## Materials and Methods

### Plant materials and trait collection

A set of 210 elite inbred rapeseed lines with abundant phenotypic variation were collected to construct an association panel (Supplementary Table S1); 55 lines (X001-X055) were used to isolate and characterize the sucrose transporter (SUT) gene^50^, and 155 lines (X056-X210) were derived from an association mapping population genotyped using the 60 K Illumina^®^ Infinium SNP array^51^. The yield-related traits of these lines were measured in a previous study^50^,^51^. The 210 lines were grown with 20 replications in 10 × 10 culture plates. When cotyledons were fully developed, all of the lines were sprayed with nutrient solution as described previously^52^. To control environmental conditions, the seedlings were grown in an illuminated culture room under 16 L:8D conditions at 20 °C and measurements performed on day 20. Photographs of seedlings were analyzed using AutoCAD software (http://www.autodesk.com.cn/education/free-software/featured). Three long hypocotyl (L) and three short hypocotyl (S) lines were used for transcriptome sequencing. These lines were selected from a doubled haploid (DH) population (DH-6004) developed from 2011–5515–137 × Gui01A10 F1 (field code 9–6004), in which ‘2011–5515–137’ exhibits early flower and ‘Gui01A10’ moderate flower.

### SNP genotyping

Fifty-five lines (X001-X055) and six DHs (DH1, DH2, DH3, DH4, DH5 and DH6) were genotyped using the *Brassica* 60 K Illumina ^®^ Infinium SNP array. Combined with genotype information obtained previously for the other 155 lines, 26,016 SNPs were mapped in silico using the probe sequences of 52,157 SNPs to perform a Blast N search against *B. napus* genome sequences^53^. Only the top hits, using an E-value cut-off of 1E-15 against the *B. napus* genome sequences, were considered. Hits with AA or BB frequency equal to zero (i.e., monomorphic), call frequency <0.8, or minor frequency <0.05 were excluded. Thus, a total of 23,435 SNPs were filtered for association analysis (Supplementary Table S4). Genetic diversity and Nei’s genetic distance^54^ were estimated using *PowerMarker* version 3.25^55^.

### Population structure, relative kinship, and linkage disequilibrium

The population structure was inferred using the software package *STRUCTURE* v2.3.4^56^ based on 5,334 SNPs with AA or BB frequency >0.05, call frequency ≥0.9 and minor frequency >0.2. Five independent runs were performed with a K-value (the putative number of genetic groups) from 1 to 10, with both the length of the burning period and the number of Markov Chain Monte Carlo (MCMC) replications after burning set to 100,000 iterations under the ‘admixture model’. The most likely k-value was determined by the log probability of data [LnP(D)] and ad hoc statistic Δk based on the rate of change of LnP(D) between successive k values as described previously^57^. Accessions with a probability of membership >0.7 were assigned to corresponding clusters, and those with a probability of membership <0.7 were assigned to a mixed group. The relative kinship matrix comparing all pairs of accessions was calculated using the software package *SPAGeDi*^58^. Negative values between two individuals were set to 0^59^. Principal component analysis (PCA) based on SNPs was carried out using the *EIGENSTRAT* tool^60^. The linkage disequilibrium (LD) parameter *r*^*2*^ was calculated using the software *TASSEL* 3.0 with 1,000 permutations^61^.

### GWAS and statistical analysis

The effects of population structure (Q, PC) and kinship (K) on the traits were evaluated by a GWAS using five models (GLM, Q, PCA, PCA + K, and Q + K). Significant loci were identified by comparing *P*-values with the Bonferroni threshold (0.05/23,435 = 2.13E-06). Quantile-quantile plots of the estimated –log_10_ (*P*) values in the association mapping model were created using the observed *P*-values from marker-trait associations versus the expected *P*-values. In addition, false discovery rates (FDRs) were calculated as [(*m* × *P*)/*n*] × 100%, where *m* is the total number of SNPs (23,435 in this study), *P* is the *P*-value threshold for detecting a significant association, and *n* is the total number of significant associations per trait^62^.

Phenotypic variation, correlation and linear regression analyses were performed using *SPSS* version 19.0 (IBM Corp., Armonk, NY, USA).

### Nuclear RNA extraction and RNA sequencing

When the second cotyledons were fully expanded in the illuminated culture room, the seedlings of three S lines (DH1, DH2 and DH3) and three L lines (DH4, DH5 and DH6) were pooled to long hypocotyl bulk and short hypocotyl bulk, respectively, then immediately frozen in liquid nitrogen and stored at −80 °C. Total nuclear RNA was extracted from ~100 mg of frozen plants using the RNAprep Pure Plant Kit (TIANGEB BIOTECH, Beijing, China) according to the manufacturer’s instructions in two biological replicates. NanoDrop ND 1000 (NanoDrop technologies) was used to evaluate the quality of the extracted RNA. RNA with an RNA Integrity Number (RIN) > 8 as assessed by Agilent Technologies 2100 Bioanalyzer (Agilent) was used to prepare the c-DNA library. The sequencing library was generated using the Illumina RNA Library Prep Kit (NASDAQ: ILMN, America) following the manufacturer’s recommendations. The library preparations were sequenced on an Illumina Hiseq 200 platform, and 100-bp paired-end reads were generated.

### DEG identification and gene annotations

The sequenced data were trimmed by removing adapters, low-quality sequences or bases, and contaminations or overrepresented sequences using *Trimmomatic* software version 0.33. The clean reads were aligned to the *B. napus* reference genome^22^ using *Hisat* software version 0.1.6 and then assembled using TopHat 2.0.0 and Cufflinks^63^. Fragments per kilobase million (FPKM) was determined to estimate gene expression levels. Differentially expressed genes (DEGs) between two genotypes were identified by Cuffdiff based on the criteria *P* < 0.05 and |log^2^ (L/S)| > 1. To identify possible homologous genes, DEGs were blasted against the *A. thaliana* genome database (http://www.arabidopsis.org/). The GO enrichment analysis for DEGs was implemented by *Blast2GO* and significantly enriched GO terms (*P* < 0.05) displayed using the online tool WEGO (http://wego.genomics.org.cn). The enrichment of DEGs was determined by KEGG pathway analysis using the KOBAS2.0 website (http://kobas.cbi.pku.edu.cn/home.do). To analyze the metabolic pathway and functional classification of DEGs, expression data were mapped to metabolic pathways using *MapMan* software^64^.

## Additional Information

**How to cite this article:** Luo, X. *et al*. Joint genome-wide association and transcriptome sequencing reveals a complex polygenic network underlying hypocotyl elongation in rapeseed (*Brassica napus L.*). *Sci. Rep.* **7**, 41561; doi: 10.1038/srep41561 (2017).

**Publisher's note:** Springer Nature remains neutral with regard to jurisdictional claims in published maps and institutional affiliations.

## Supplementary Material

Supplementary Information

Supplementary Table S1

Supplementary Table S4

Supplementary Table S6

Supplementary Table S9

## Figures and Tables

**Table 1:** Summary of SNPs significantly associated with hypocotyl elongation.

SNP	Chromosome	Allele	Position	MAF	*P*-value	R^2^(%)
Bn-scaff_16110_1-p587456	C07	A/G	44303399	0.49	1.94E-06	4.82
Bn-scaff_16110_1-p670992	C07	G/A	44218661	0.50	7.25E-07	
Bn-scaff_16110_1-p671315	C07	A/G	44218337	0.49	7.66E-07	
Bn-scaff_16110_1-p685258	C07	A/G	44204592	0.50	6.87E-07	
Bn-scaff_16110_1-p685428	C07	C/A	44204201	0.50	7.24E-07	

MAF, minor allele frequency; R^2^(%), amount of phenotypic variation for each trait explained by multiple SNPs.

**Table 2:** Differentially expressed genes detected by transcriptome sequencing and genome-wide association.

*B. napus*					*A. thaliana*	
Gene ID	Position	log2(L/S)	*P*-value	L change	Homologous	Description
*BnaC07g45310D*	43459847	1.957	0.001	Up	*AT4G34980*	Serine protease similar to subtilisin
*BnaC07g45430D*	43513273	6.841	0	Up	*—*	
*BnaC07g45440D*	43518096	3.658	0	Up	*AT4G35160*	Encodes a cytosolic N-acetylserotonin O-methyltransferase
*BnaC07g45500D*	43543746	−1.789	0.038	Down	*AT4G35270*	Plant regulator RWP-RK family protein
*BnaC07g45520D*	43560727	5.151	0	Up	*AT4G35310*	calmodulin-domain protein kinase
*BnaC07g45590D*	43590520	2.228	0	Up	*AT4G35460*	NADPH-dependent thioredoxin reductase 1
*BnaC07g45710D*	43643363	−1.57	0.033	Down	*AT4G35600*	Encodes a receptor-like cytoplasmic kinase
*BnaC07g45720D*	43646363	−4.048	0	Down	*—*	
*BnaC07g46060D*	43817797	1.646	0.029	Up	*AT4G36250*	Encodes a putative aldehyde dehydrogenase
*BnaC07g46090D*	43890978	2.268	0.005	Up	*AT4G36620*	Encodes a member of the GATA factor family of zinc finger transcription factors
*BnaC07g46260D*	43972670	3.074	0.017	Up	*AT4G36820*	Protein of unknown function
*BnaC07g46560D*	44131930	−1.645	0.013	Down	*AT4G37530*	Peroxidase superfamily protein
*BnaC07g46630D*	44164232	−2.414	0	Down	*AT4G37610*	BTB and TAZ domain protein
*BnaC07g46660D*	44180768	−1.175	0.044	Down	*AT4G37640*	Encodes a calmodulin-regulated Ca(2+)-pump
*BnaC07g46770D*	44253480	−1.725	0.037	Down	*AT4G37750*	Encodes a putative transcriptional regulator similar to AP2
					*AT4G36920 (AP2)*	Encodes a floral homeotic gene
*BnaC07g46780D*	44290014	1.138	0.02	Up	*AT4G37800*	Xyloglucan endotransglucosylase/hydrolase 7
*BnaC07g46820D*	44311339	1.123	0.038	Up	*AT4G37890*	Embryo sac development arrest 40
*BnaC07g46830D*	44375293	−1.362	0.028	Down	*AT4G39850*	Encodes a peroxisomal protein of the ATP binding cassette
*BnaC07g46910D*	44433889	−4.869	0	Down	*AT4G39830*	Cupredoxin superfamily protein
*BnaC07g46940D*	44454217	1.424	0.016	Up	*AT4G39780*	Protein contains one AP2 domain
*BnaC07g47090D*	44497714	−4.227	0	Down	*AT4G39600*	Galactose oxidase/kelch repeat superfamily protein
*BnaC07g47300D*	44591928	−1.42	0.046	Down	*AT4G39240*	Galactose oxidase/kelch repeat superfamily protein
*BnaC07g47340D*	44603110	1.861	0.004	Up	*AT4G39200*	Ribosomal protein S25 family protein
*BnaC07g47370D*	44615843	−2.426	0.042	Down	*AT4G39160*	Homeodomain-like superfamily protein
*BnaC07g47430D*	44647393	2.13	0.002	Up	*—*	
*BnaC07g47440D*	44649385	1.179	0.044	Up	*—*	
*BnaC07g47450D*	44661277	3.187	0.001	Up	*AT4G39010*	Glycosyl hydrolase 9B18
*BnaC07g47470D*	44670819	1.54	0.004	Up	*AT4G38970*	Response to ABA
*BnaC07g47720D*	44768381	−5.589	0	Down	*AT4G38540*	FAD/NAD(P)-binding oxidoreductase family protein

A dash indicates no homolog has been identified in the respective genome.
