# Supplementary material for: Joint genome-wide association and transcriptome sequencing reveals a complex polygenic network underlying hypocotyl elongation in rapeseed (Brassica napus L.)
Source: Sci Rep. 2017 Jan 31;7:41561. doi: 10.1038/srep41561 (PMC5282501; doi:10.1038/srep41561)
Supplement: Supplementary Information [file srep41561-s1.doc]

**Joint genome-wide association and transcriptome sequencing reveals a complex polygenic network underlying** **hypocotyl elongation in rapeseed (*Brassica napus L.*)**

**Xiang Luo, Zhifei Xue, Chaozhi Ma, Kaining Hu, Ziru Zeng, Shengwei Dou, Jinxing Tu, Jinxiong Shen, Bin Yi & Tingdong Fu**

National Key Laboratory of Crop Genetic Improvement, National Center of Rapeseed Improvement in Wuhan, Huazhong Agricultural University, Wuhan 430070, P.R. China.

Correspondence and requests for materials should be addressed to C.M

E-mail: [yuanbeauty@mail.hzau.edu.cn](mailto:yuanbeauty@mail.hzau.edu.cn)

National Key Laboratory of Crop Genetic Improvement, National Center of Rapeseed Improvement in Wuhan, Huazhong Agricultural University, Wuhan 430070, P.R. China.

Tel. +86-27-87281676.

Fax. +86-27-87280009.

**Table S2. Phenotypic variation for hypocotyl elongation of 210 inbred accessions.**

| Trait | Mean Ậ+SD^a^ | Range | Skew | Kurt | CV﹪^b^ |
| --- | --- | --- | --- | --- | --- |
| Hypocotyl  elongation | 2.66+0.44 | 1.38-4.81 | 0.53 | 0.75 | 20.93 |

^a^ SD: standard deviation

^b^ CV(﹪): coefficient of variation

**Table S3. Correlation coefficients between hypocotyl elongation and yield-related traits.**

| Trait | ^a^SP | ^a^SS | ^a^BLY | ^a^TSW | ^b^PH | ^c^BN | ^b^SY | ^c^BY | ^b^HI |
| --- | --- | --- | --- | --- | --- | --- | --- | --- | --- |
| Hypocotyl elongation | -0.16 | -0.16 | 0.04 | -0.10 | 0.19* | 0.06 | 0.29** | 0.21** | -0.03 |

SP, numbers of siliques per plant; SS, number of seeds per silique; BLY, block yield; TSW, thousand seed weight; PH, plant height; BN, branch number; SY, seed yield per plant; BY, biomass yield per plant; HI, harvest index.

^a^ Li et al. 2011

^b^ Luo et al. 2015

^C^ Common trait in both papers. The corrected value as the difference between the sample value and corresponding population.

^*^ *P* = 0.05, ^* *^ *P* = 0.01.

**Table S5. Estimated LD decay for each chromosome.**

| Chromosome | Number of SNPs | SNP density | LD decay (kb) |
| --- | --- | --- | --- |
| A01 | 1010 | 23.04 | 506.61 |
| A02 | 708 | 35.02 | 757.08 |
| A03 | 1466 | 20.31 | 495.13 |
| A04 | 976 | 19.62 | 559.00 |
| A05 | 1083 | 21.30 | 530.60 |
| A06 | 1034 | 23.59 | 576.15 |
| A07 | 1335 | 17.98 | 459.03 |
| A08 | 764 | 24.82 | 560.97 |
| A09 | 1075 | 31.50 | 968.17 |
| A10 | 1014 | 17.16 | 488.66 |
| C01 | 1913 | 20.30 | 886.03 |
| C02 | 1777 | 26.01 | 938.46 |
| C03 | 2120 | 28.57 | 1195.66 |
| C04 | 2575 | 19.00 | 970.42 |
| C05 | 641 | 67.37 | 1627.32 |
| C06 | 921 | 40.42 | 914.92 |
| C07 | 1244 | 35.99 | 754.95 |
| C08 | 1148 | 33.52 | 602.91 |
| C09 | 631 | 76.88 | 3190.79 |

LD decay values are the genetic distance at which *r*^2^ fell below 0.1.

**Table S7. Summary of RNA-Seq reads.**

|  | S1 | S2 | L1 | L2 |
| --- | --- | --- | --- | --- |
| No. of total raw reads | 26812972 | 54069011 | 55426510 | 28874038 |
| No. of clean reads | 23605571 | 49167142 | 49967154 | 25879960 |
| No. of unique match reads | 14487343 | 32395410 | 33856956 | 15189970 |
| No. of multiple match reads | 3194033 | 7074201 | 3961808 | 3237090 |
| Rate of aligned pairs | 79.42% | 86.49% | 77.69% | 73.09% |

**Table S8. Gene Ontology (GO) enrichment analysis of differentially expressed genes.**

| Go:ID | Function | Total number | Genes |  |  |  |  |  |  |  |  |  |  |
| --- | --- | --- | --- | --- | --- | --- | --- | --- | --- | --- | --- | --- | --- |
| GO:0009725 | Response to hormone | 6 | BnaC07g45520D, BnaC07g47470D, BnaC07g45710D,  BnaC07g45720D, BnaC07g46630D, BnaC07g46660D | | | | | | | | | | |
| GO:0009755 | Hormone-mediated signaling pathway | 3 | BnaC07g45520D, BnaC07g45710D, BnaC07g45720D | | | | | |  |  |  |  |  |
| GO:0032870 | Cellular response to hormone stimulus | 3 | BnaC07g45520D, BnaC07g45710D, BnaC07g45720D | | | | | |  |  |  |  |  |
| GO:0048439 | Flower morphogenesis | 2 | BnaC07g46630D, BnaC07g46770D | | | |  |  |  |  |  |  |  |
| GO:0009908 | Flower development | 3 | BnaC07g46090D, BnaC07g46630D, BnaC07g46770D |  |  |  |  |  |  |  |  |  |  |


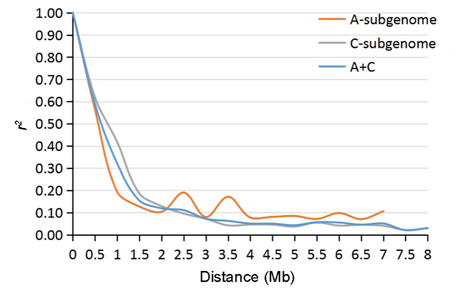


**Figure S1**. **LD analysis across the subgenome.**


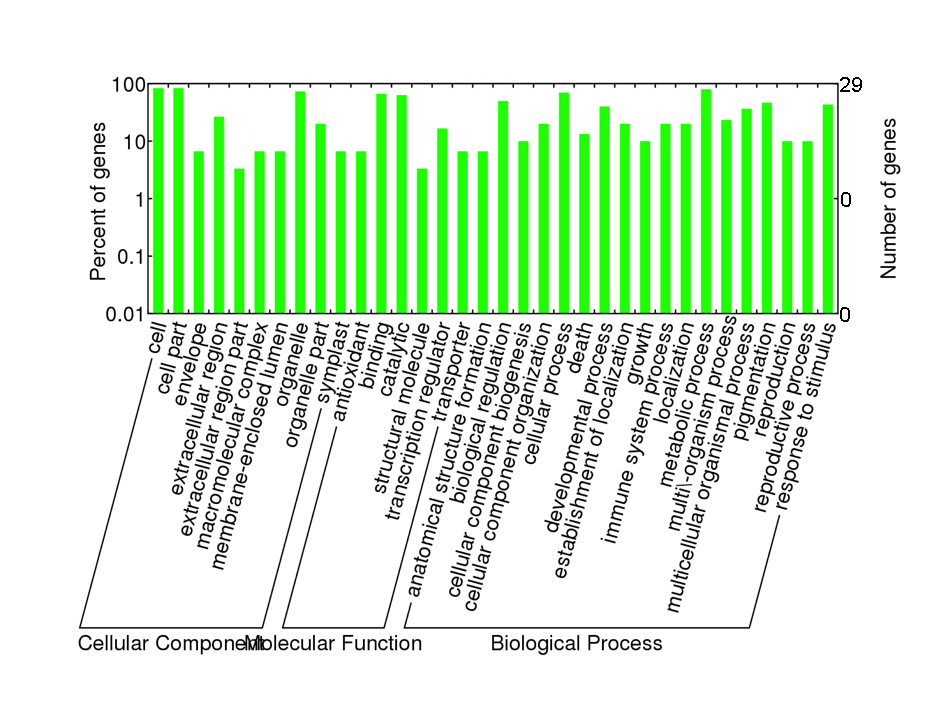


**Figure S2.** **GO analysis of the 29 candidate genes detected in the GWAS followed by transcriptome analysis.**

Figure Legends

**Figure 1.** **Phenotypic variation in hypocotyl length.** (A) Frequency of phenotypic variation in 210 accessions. (B) Comparison of two phenotypes by *t*-test.

**Figure 2. Analysis of the population structure of 210 rapeseed accessions using *STRUCTURE*.** (A) Estimated L(K) of possible clusters (k) from 1 to 10. (B) Delta K based on the rate of change of L(K) between successive K values. (C) Population structure based on k=2. Red represents subgroup Q1; green represents subgroup Q2. (D) Principal component (PC) analysis.

**Figure 3. Genetic distance and kinship coefficient analysis between pairs of accessions.**

**Figure 4. Association analyses of hypocotyl elongation.** (A) Quantile–quantile plots of estimated −log_10_(*P*) from the association analysis of hypocotyl elongation. The black line represents expected *P*-values with no associations. The red line represents observed *P*-values using the GLM model. The green line represents observed *P*-values using the Q model. The blue line represents observed *P*-values using the PCA model. The cyan line represents observed *P*-values using the Q+K model. The pink line represents observed *P*-values using the PCA+K model (color figure online). (B) Manhattan and quantile–quantile plots generated from the genome-wide association analysis of hypocotyl elongation. The blue horizontal line depicts the Bonferroni significance threshold (2.13 E-6).

**Figure 5. LD and haplotype analysis for five associated SNPs.** (A) Association peak on chromosome C07. (B) LD analysis among the associated SNPs. (C) Haplotype analysis with associated SNPs in the population. (D) Phenotypic variation of hypocotyl length in each haplotype.

**Figure 6.** **The distribution pattern of candidate genes and SNPs associated with hypocotyl elongation.** The abbreviations for orthologous genes in *A. thaliana* are shown in parentheses. SNPs are marked in red. Numbers represent the relative distance in the reference genome in kilobases.
